# Chemical Composition, Free Radicals and Pathogenic Microbes in the Extract Derived from *Dictyota dichotoma*: In Silico and In Vitro Approaches

**DOI:** 10.3390/md22120565

**Published:** 2024-12-17

**Authors:** Fouad Oumassi, Khalid Chebbac, Naouar Ben Ali, Soundouss Kaabi, Zineb Nejjar El Ansari, Amira Metouekel, Azeddin El Barnossi, Abdelfattah El Moussaoui, Mohamed Chebaibi, Loubna Bounab, Ibrahim Mssillou, Abdelaaty Abdelaziz Shahat, Brahim El Bouzdoudi, Mohammed L’bachir El Kbiach

**Affiliations:** 1 Plant Biotechnology Team, Faculty of Sciences, Abdelmalek Essaadi University, Tetouan 93002, Morocco; benalinaouar@yahoo.fr (N.B.A.); soundouss.kaabi@um.es (S.K.); zainabnejjar@gmail.com (Z.N.E.A.); a.elmoussaoui@uae.ac.ma (A.E.M.); belbouzdoudi@uae.ac.ma (B.E.B.); melkbiach@uae.ac.ma (M.L.E.K.); 2 Laboratory of Biotechnology and Preservation of Natural Resources, Faculty of Sciences Dhar El Mahraz, Sidi Mohammed Ben Abdallah University, Fez 30000, Morocco; khalid.chebbac@usmba.ac.ma; 3 Department of Plant Biology (Plant Physiology), Faculty of Biology, University of Murcia, 30100 Murcia, Spain; 4 Life and Health Sciences Team, Faculty of Medicine and Pharmacy, Abdelmalek Essaadi University, Tetouan 93000, Morocco; 5 Laboratoire R&D BOI, Bioval Océan Indien Research and Innovation Company, 18 rue des Poivres Roses, 97419 La Possession, Reunion Island, France; metouekel.amira@gmail.com; 6 University of Technology of Compiegne, EA 4297 TIMR, CEDEX, 60205 Compiegne, France; 7 Biological Engineering Laboratory, Faculty of Sciences and Techniques, Sultan Moulay Slimane University, Beni Mellal 23000, Morocco; azeddin.elbarnossi@usmba.ac.ma; 8 Ministry of Health and Social Protection, Higher Institute of Nursing Professions and Health Techniques, Fez 30000, Morocco; mohamed.chebaibi@yahoo.fr; 9 Advanced Materials, Structures and Civil Engineering Team, ENSA Tetouan, Abdelmalek Essaadi University, Tetouan 93000, Morocco; lbounab@uae.ac.ma; 10 Laboratory of Natural Substances, Pharmacology, Environment, Modeling, Health & Quality of Life (SNAMOPEQ), Faculty of Sciences Dhar El Mahraz, Sidi Mohamed Ben Abdellah University, Fez 30000, Morocco; 11 Pharmacognosy Department, College of Pharmacy, King Saud University, Riyadh 11451, Saudi Arabia; ashahat@ksu.edu.sa

**Keywords:** *Dictyota dichotoma*, extract, HPLC-MS, DPPH, antimicrobial

## Abstract

Marine algae are renowned for their health benefits due to the presence of functional bioactive compounds. In this context, this study aims to valorize the extract of a seaweed, *Dictyota dichotoma* (*D. dichotoma*), through phytochemical characterization using liquid chromatography–mass spectrometry (HPLC-MS), as well as in vitro and in silico evaluation of its biological activities (antioxidant and antimicrobial). Phytochemical characterization revealed that the ethanolic extract of *Dictyota dichotoma* (DdEx) is rich in phenolic compounds, with a total of 22 phycocompounds identified. Antioxidant activity, measured by various methods, showed an IC_50_ of 120 µg/mL for the DPPH assay, an EC_50_ of 120.53 µg/mL for the FRAP assay, and a total antioxidant power of 685.26 µg AAE/mg according to the phosphomolybdate (TAC) method. Evaluation of antibacterial activity showed a zone of inhibition diameter ranging from 11.93 to 22.58 mm, with the largest zone observed for the *Escherichia coli* (*E. coli*) strain. For antifungal activity, inhibition zone diameters ranged from 22.38 to 23.52 mm, with the largest recorded for the *Saccharomyces cerevisiae* (*S. cerevisiae*) strain. The in silico study identified tetragalloyl-glucose, apigenin-7-O-glucoside, and pentagalloyl-glucose as the most active compounds against NADPH oxidase, with docking scores of −7.723, −7.424, and −6.402 kcal/mol, respectively. Regarding antibacterial activity, apigenin-7-O-glucoside, pelargonidin-3-O-glucoside, and secoisolariciresinol demonstrated high affinity for *E. coli* beta-ketoacyl-[acyl carrier protein] synthase, with docking scores of −7.276, −6.811, and −6.594 kcal/mol, respectively. These in vitro and in silico evaluations showed that *D. dichotoma* extract possesses antioxidant and antimicrobial properties, due to its richness in bioactive compounds identified by HPLC.

## 1. Introduction

The Mediterranean Sea is recognized as a marine biodiversity hotspot, home to around 17,000 species, nearly 20% of which are endemic, thanks to its semi-enclosed geographical location [1,2]. Among this wealth, the Mediterranean basin boasts 1124 species of algae [3]. As the main biomass producers in marine ecosystems, seaweeds produce a wide range of bioactive compounds with antibacterial, antifungal, antimacrofouling and other therapeutic properties [4,5,6]. Although widely consumed in Asia, seaweed has also found applications in industry, notably for the production of agar, alginate and carrageenan [7]. Present in a variety of shapes, colors and sizes, algae colonize mainly rocky and shallow coastal areas, especially at low tide [8]. For millennia, coastal populations have harvested and consumed them as sea vegetables [9]. They also play a crucial role as a habitat for many aquatic species, providing food, shelter and vegetation. Depending on their pigments, morphology and reproductive characteristics, seaweeds are classified into different groups: Phylum Chlorophyta (Class Ulvophyceae), Phylum Rhodophyta and Phylum Ochrophyta (Class Phaeophyceae) [10].

Macroalgae live in extreme environments, with variations in several factors. They have to adapt rapidly to the occurring changes, while producing primary metabolites that act as a deterrent against stress. In addition, the production of secondary metabolites responds to changes occurring in the environment [11]. Bioactive molecules derived from algae are widely used in pharmacology, biomedicine, microbiology, agriculture and molecular biology. Recently, seaweed-derived phycocompounds have received a great deal of attention, particularly in the pharmaceutical industry. Seaweeds contain numerous clinically important bioactive compounds with antioxidant, anticancer, antimicrobial, anti-inflammatory, antiviral and antitumor activities, as well as neuroprotective properties [12,13]. In addition, multiple processes are used, such as fermentation, anaerobic digestion, liquefaction, transesterification and thermochemical conversion to transform algae biomass into high-value energy products, including biomethane, bioethanol, biogas, biofuels and bio-oils [13].

Obesity, digestive disease, cardiovascular disease (such as hypertension, stroke and myocardial infarction), certain types of cancer and osteoporosis are all food-related diseases. The abundance of disease-fighting bioactives, such as polysaccharides (alginate, fucoidan, agar and carrageenan), polyphenols, natural pigments (fucoxanthin, chlorophyll, β-carotene, xanthophyll), vitamins, proteins and minerals in algae, enables them to be used as human food or as raw materials for various valuable natural products of nutritional interest [11,14].

The *Dictyota* genus, belonging to the Dictyotaceae family, is distinguished by its importance in coastal areas, where it is one of the most significant brown algae. Its high biomass and wide geographical distribution in temperate and tropical seas make it an essential component of marine ecosystems. Species in this family are particularly rich in secondary metabolites, with a predominance of diterpenes, compounds recognized for their diverse biological activities, including antimicrobial, anti-inflammatory and antitumor activity. Despite the popularity of brown algae, including *Dictyota*, in traditional practices and certain industries, their potential in phytopharmacology remains largely underexploited. Scientific studies on the therapeutic properties of *Dictyota* species remain limited, suggesting a vast field of research for the identification of new bioactive compounds and the development of innovative therapeutic products [15,16]. *Dictyota dichotoma*, the type species of the genus, plays a key role as a taxonomic reference, underlining its importance in the study and classification of brown algae [17]. Future research on this species could reveal promising applications in pharmacology and contribute to a better understanding of marine biodiversity and its exploitable resources.

The aim of this study was to explore the therapeutic potential of *Dictyota dichotoma* seaweed extract, focusing on its antioxidant and antibacterial properties. To this end, a detailed phytochemical characterization of the extract was carried out using liquid chromatography–mass spectrometry (HPLC-MS), in order to identify the main bioactive compounds. Biological activities will then be assessed using complementary approaches, including in vitro tests to determine antioxidant and antibacterial capacities, and in silico simulations to predict molecular interactions and better understand potential mechanisms of action. This integrated approach will highlight the potential applications of *Dictyota dichotoma* in the development of new natural therapeutic solutions.

## 2. Results

### 2.1. Phytochemical Characterisation by HPLC-MS

**Figure 1:**
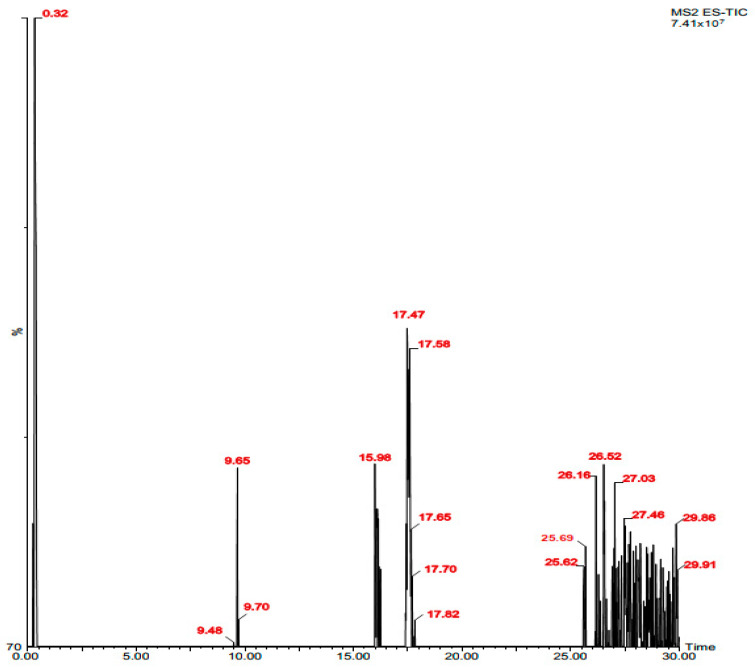
Chromatographic profile of phycocompounds identified in *Dictyota dichotoma* extract by HPLC-MS.

Phytochemical analysis by HPLC of the ethanolic extract of the alga *D. dichotoma* reveals a wealth of polyphenolic compounds, mainly hydrolysable tannins and flavonoids (Figure 1 and Table 1).

### 2.2. Antioxidant Activity

**Figure 2:**
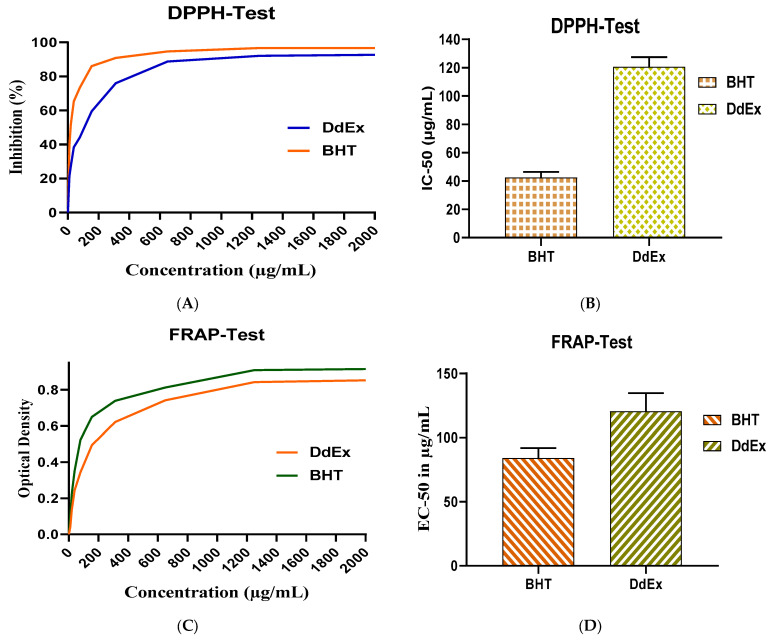

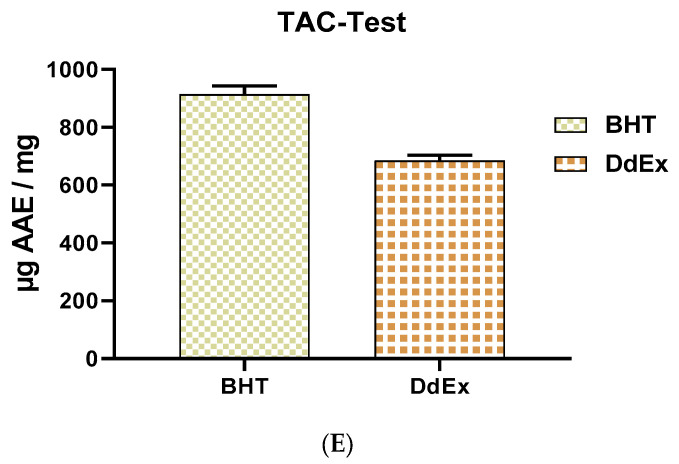
Assessment of antioxidant power using the DPPH method (**A**,**B**), the FARP method (**C**,**D**) and the phosphomolybdate (TAC) method (**E**).

To further assess the antioxidant capacity of the extract, three methods were used: the DPPH-free radical scavenging method (DPPH), reducing power test (FRAP) and total antioxidant capacity (TAC), with butylated hydroxytoluene (BHT) used as a reference. The ethanolic extract of *D. dichotoma* (DdEx) showed dose-dependent antioxidant activity using the DPPH method. At a concentration of 10 µg/mL, DdEx inhibited 21.36% of free radicals, compared with 39.47% for BHT. This antioxidant activity increased with concentration, reaching 94.09% inhibition at 2000 µg/mL, close to the 97.73% observed for BHT (Figure 2A). Although DdEx is less effective at low concentrations, it shows competitive activity at higher concentrations, suggesting a promising antioxidant potential despite the need for higher doses compared with BHT. Figure 2B shows the semi-maximal inhibitory concentration (IC_50_). According to these results, the IC_50_ of the extract (120 µg/mL) is higher than that of BHT (40 µg/mL). Phytochemical screening revealed the presence of phenolic compounds, which could explain this antioxidant activity.

The results illustrated in Figure 2C,D, show that *D. dichotoma* extract possesses a notable capacity to reduce the ferric-tripyridyltriazine complex (Fe^3+^-TPTZ) to the ferrous-tripyridyltriazine complex (Fe^2+^-TPTZ). Evaluation of the antioxidant activity of the ethanolic extract of *D. dichotoma* (DdEx) by the FRAP method revealed a dose-dependent reducing capacity. At low concentrations (10 µg/mL), DdEx exhibits a reducing power measured as an optical density (OD) of 0.0415, significantly lower than that of BHT, which achieves an OD of 0.1075. This difference reflects the lower initial reducing efficiency of DdEx. However, with increasing concentration, a gradual improvement in the reducing power of DdEx is observed. At a concentration of 2000 µg/mL, the OD reaches 0.8685, indicating significant activity, although still slightly lower than that of BHT, which reaches an OD of 0.925 at the same concentration. These results (Figure 2C) demonstrate a positive correlation between DdEx concentration and its reducing activity, although it remains overall lower than that of BHT, a reference antioxidant. These findings confirm the antioxidant potential of DdEx while highlighting its relative potency compared to BHT.

These results suggest that, although the antioxidant potential of the extract increases with increasing concentration, its efficacy remains lower than that of BHT, probably due to the lower reducing power of the active compounds or their lower concentration in the extract. The EC_50_ (effective concentration to obtain 50% of the maximum effect) is a key measure for evaluating the potency of an antioxidant. In this case, the EC_50_ of BHT is 84.13 µg/mL, while that of the ethanolic extract of *D. dichotoma* (DdEx) is 120.53 µg/mL (Figure 2D). This indicates that BHT is more potent than DdEx, requiring a lower concentration to achieve 50% of its maximum antioxidant effect. In contrast, DdEx, with a higher EC_50_, is less effective, requiring a higher concentration to achieve a similar effect. These results suggest that although DdEx has antioxidant potential, it is less potent than BHT.

Evaluation of the total antioxidant capacity (TAC) shows that BHT has a higher antioxidant capacity than the ethanolic extract of *D. dichotoma* (DdEx). BHT has a value of 914.932 µg AAE/mg, while DdEx has a value of 685.265 µg AAE/mg (Figure 2E). This difference indicates that BHT has a greater capacity to neutralise free radicals than DdEx. The results suggest that although DdEx has significant antioxidant potential, it remains less effective than BHT in this test, which may be linked to the nature of the antioxidant compounds present in the extract.

### 2.3. Antibacterial and Antifungal Activity of D. dichotoma Extract

The results of the antimicrobial potency of *D. dichotoma* extract, including the inhibition diameter (ID), are summarised in Table 2. *D. dichotoma* algae showed antifungal promoting effects against both yeasts with a maximum inhibition zone of 23.52 ± 0.93 mm on *Saccharomyces cerevisiae* (*S. cerevisiae*) and 22.38 ± 1.24 mm on *Candida albicans* (*C. albicans*). In addition, significant antibacterial activity was demonstrated against all tested bacterial strains, including Gram-negative (*Escherichia coli* (*E. coli*), *Proteus mirabilis* (*P. mirabilis*) and *Klebsiella pneumoniae* (*K. pneumoniae*)) bacteria with inhibition zones of 22.58 ± 0.58, 18.81 ± 1.05 and 10.56 ± 1.48 mm, respectively, and Gram-positive bacteria (*Staphylococcus aureus* (*S. aureus*)) with an inhibition zone of 11.93 ± 0.73 mm. Although these microbes are known to be very virulent and pathogenic, they have been shown to be susceptible to the alga *D. dichotoma*.

### 2.4. Molecular Docking

NADPH oxidase is an essential enzyme responsible for the formation of reactive oxygen species (ROS), with its activity being tightly regulated within cells. While ROS produced by NADPH oxidase are vital for pathogen defense and cellular signaling, excessive ROS can result in oxidative stress, contributing to various diseases. Inhibiting NADPH oxidase is a potential strategy to enhance antioxidant activity and protect against oxidative stress-related conditions. By reducing ROS production, enhancing cellular antioxidant defenses, and decreasing chronic inflammation, NADPH oxidase inhibitors may play a crucial role in maintaining cellular health and preventing disease progression. In our in silico study, tetragalloyl-glucose, apigenin-7-O-glucoside and pentagalloylglucose were the most effective molecules against NADPH oxidase, with glide gscores of −7.723, −7.424 and −6.402 kcal/mol, respectively (Table 3). Regarding antibacterial activity, apigenin-7-O-glucoside, pelargonidin 3-O-glucoside, and secoisolariciresinol were the most potent against beta-ketoacyl-[acyl carrier protein] synthase from *E. coli*, with glide gscores of −7.276, −6.811, and −6.594 kcal/mol. Additionally pentagalloylglucose, tetragalloyl-glucose, and ellagic acid were the most effective against *S. aureus* nucleoside diphosphate kinase, with glide gscores of −10.502, −10.47, and −7.935 kcal/mol (Table 3). Regarding antifungal activity, apigenin-7-O-glucoside, pelargonidin 3-O-glucoside and tetragalloyl-glucose were the most active molecules against sterol 14-alpha demethylase (CYP51) from *C. albicans* with a glide gscore of −9.569, −8.925, and −8.778 kcal/mol (Table 3).

**Figure 3:**
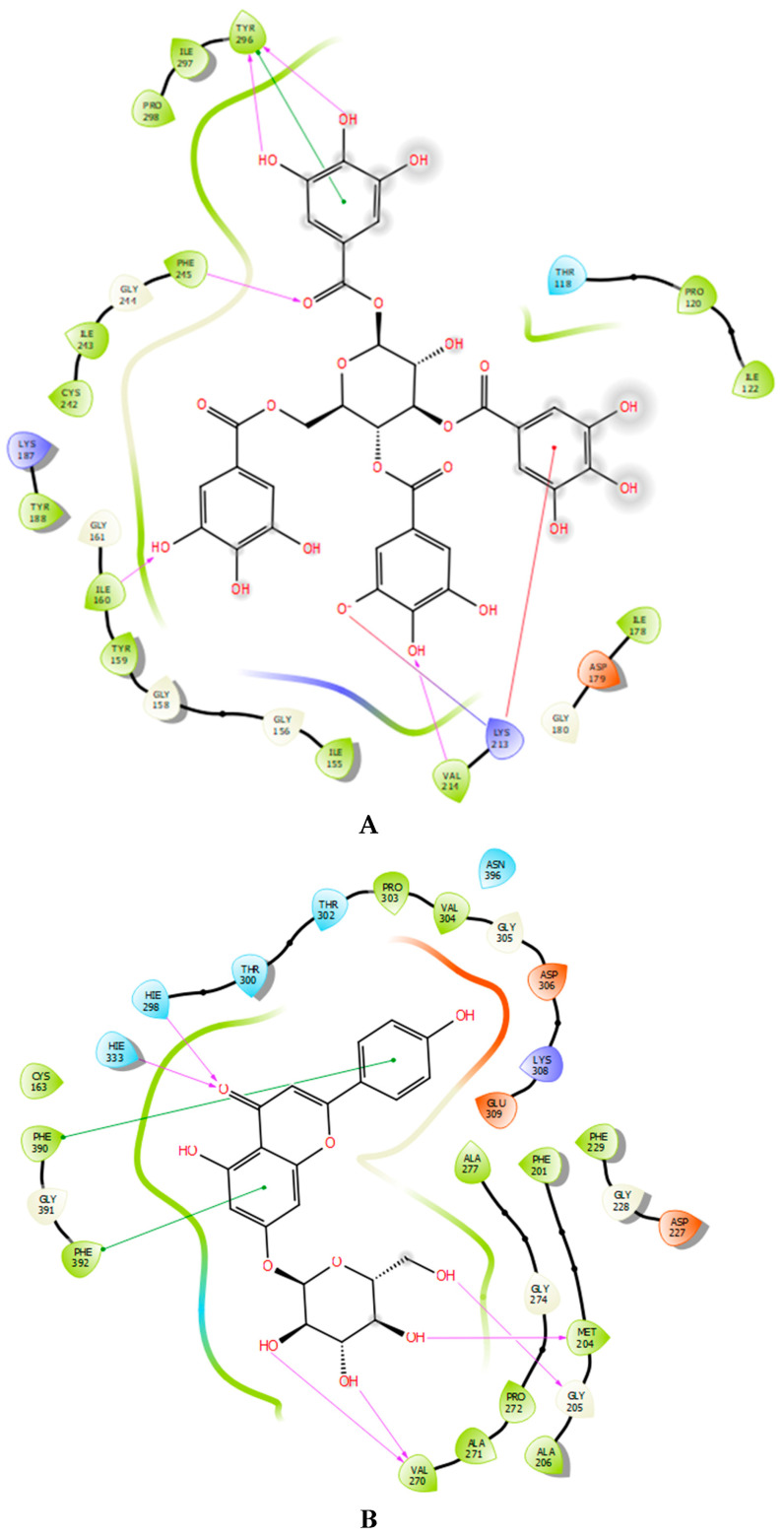

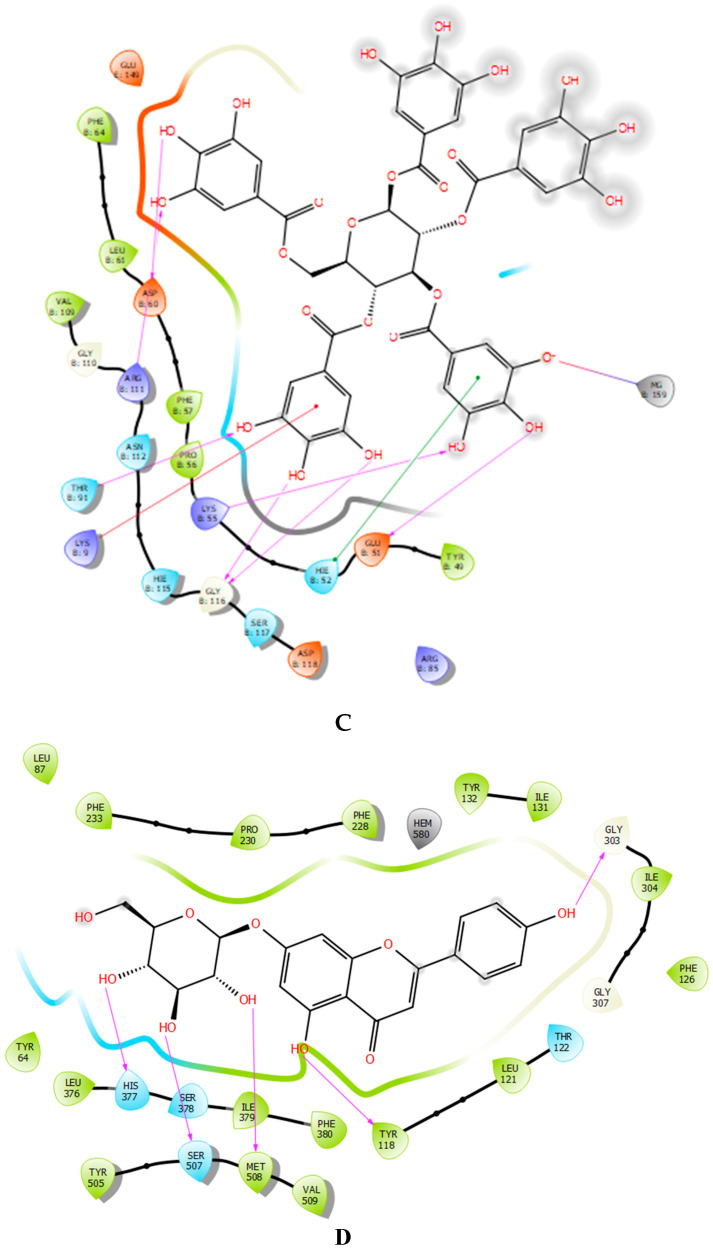
The 2D viewer of ligand interactions with the active site. (**A**): Tetragalloyl-glucose interactions with active site of NADPH oxidase. (**B**,**D**): Apigenin-7-O-glucoside interactions with beta-ketoacyl-[acyl carrier protein] synthase from *E. coli* and sterol 14-alpha demethylase (CYP51) from *C. albicans* active sites. (**C**): Pentagalloylglucose interactions with active site *S. aureus* nucleoside diphosphate kinase.

**Figure 4:**
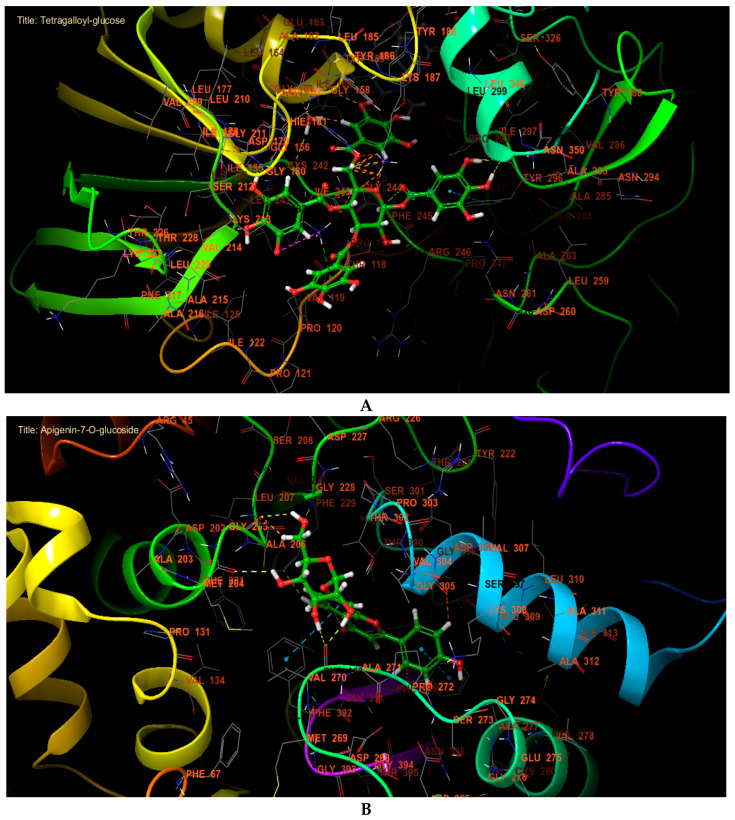

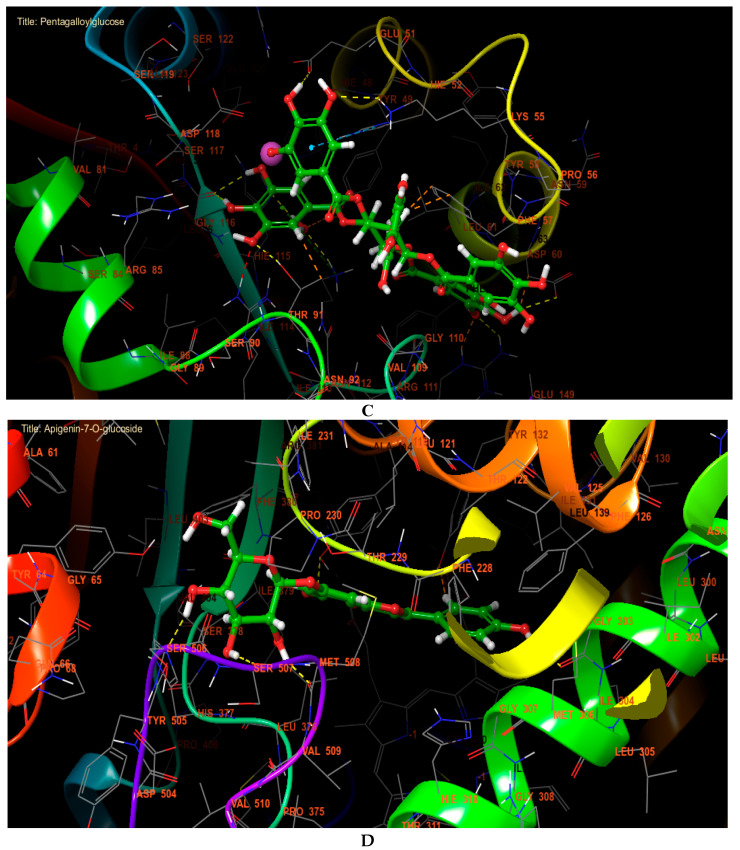
The 3D viewer of ligand interactions with the active site. (**A**): Tetragalloyl-glucose interactions with active site of NADPH oxidase. (**B**,**D**): Apigenin-7-O-glucoside interactions with beta-ketoacyl-[acyl carrier protein] synthase from *E. coli* and sterol 14-alpha demethylase (CYP51) from *C. albicans* active sites. (**C**): Pentagalloylglucose interactions with active site *S. aureus* nucleoside diphosphate kinase.

In the active site of NADPH oxidase, tetragalloyl-glucose has established five hydrogen bonds with residues TYR 296, PHE 245, ILE 160, and VAL 214 and one Pi cation bond with residue LYS 213, one Pi-Pi stacking bond with residue TYR 296, and one salt bridge with residue LYS 213 (Figure 3A and Figure 4A). However, apigenin-7-O-glucoside established six hydrogen bonds with residues HIE 298, HIE 333, VAL 270, GLY 205, and MET 204 and two Pi-Pi stacking bonds with residues PHE 390 and PHE 392 in the active site of beta-ketoacyl-[acyl carrier protein] synthase from *Escherichia coli* (Figure 3B and Figure 4B). In the active site of *S. aureus* nucleoside diphosphate kinase, pentagalloylglucose established eight hydrogen bond with residues ASP B: 60, ARG B: 111, THR B: 91, LYS B: 9, LYS B: 55, GLY B: 116, and GLU B: 51 and one Pi cation bond with residue LYS B: 9, one Pi-Pi stacking bond with residue HIE B: 52 and one salt bridge with residue MG B: 159 (Figure 3C and Figure 4C). Furthermore, apigenin-7-O-glucoside established five hydrogen bonds with the residues GLY 303, HIS 377, SER 507, MET 508, and TYR 118 in the active site of sterol 14-alpha demethylase (CYP51) from *C. albicans* (Figure 3D and Figure 4D).

## 3. Discussion

Recent studies evaluating the antioxidant activity (DPPH, FRAP, and TAC) of *D. dichotoma* phenolic compounds reveal a positive correlation between concentration and antioxidant efficiency [18]. For DPPH activity, values increase from 15.07 ± 0.88 mg/mL to 51.13 ± 2.31 mg/mL, indicating an increasing ability of the compounds to neutralize free radicals, although a plateau is observed at higher concentrations [18]. For the FRAP method, values range from 4.26 ± 0.19 mg/mL to 18.66 ± 0.84 mg/mL, demonstrating a significant improvement in the compounds’ ability to reduce ferric ions to ferrous ions [18]. Finally, the TAC method shows a notable increase from 16.27 ± 0.73 mg/mL to 71.76 ± 3.23 mg/mL, confirming the role of phenolic compounds in overall antioxidant activity, due to their ability to donate electrons or protons to neutralize free radicals. This study highlights a clear dose–response relationship for all three methods, underlining the importance of phenolic compounds in the antioxidant activities of this alga [18].

Among the phycocompounds identified by phytochemical analysis using HPLC, HHDP-digalloyl-glucose, galloyl-glucoside and tetragalloyl-glucose are particularly representative of hydrolysable tannins, known for their powerful antioxidant properties [19,20]. The presence of ellagic acid and its derivatives, such as ellagic-pentose acid and its methylated and sulphated forms, reinforces this antioxidant activity, due to their ability to neutralise free radicals [21]. Secoisolariciresinol, a lignan, and flavonoid compounds, such as apigenin-7-O-glucoside and catechin, add to the complexity of the extract with their own bilogenic properties [22,23]. The identification of these compounds suggests that *D. dichotoma* extract has considerable therapeutic potential, justifying the exploration of in vitro and in silico applications and biological evaluations as preliminary tests. These results also highlight the importance of further in-depth research into the specific biological properties of these compounds.

Antioxidants are substances that reduce oxidative stress in the human body, caused by reactive oxygen species, reactive nitrogen species and free radicals. Oxidative stress damages biological macromolecules and can lead to a variety of diseases, including cancer, stroke and cardiovascular disease. Due to the existence of eight interconnected polyphenol rings, marine algae are known to contain several bioactive compounds with potentially higher antioxidant activity than land plants [24].

The results of antioxidant tests carried out on the ethanolic extract of *D. dichotoma* (DdEx) reveal significant links with the compounds identified in the extract. The antioxidant activity measured by the DPPH test, which shows an increase in inhibition with concentration, can be attributed to the presence of phenolic compounds such as tetragalloyl-glucose and pentagalloyl-glucose. These galloyl derivatives are known for their ability to neutralise free radicals, which explains the increasing antioxidant effect observed. In addition, the results of the FRAP test, which assesses the extract’s reducing capacity, are probably influenced by compounds such as HHDP-digalloyl-glucose and tetragalloyl-glucose, known for their ability to reduce ferric complexes to ferrous [20].

The total antioxidant capacity (TAC), which reflects the overall capacity of the extract to neutralise free radicals, is largely due to the combination of several phenolic compounds, notably pentagalloyl-glucose and galloyl-HHDP-glucoside. These compounds play a crucial role in the overall antioxidant activity of the extract, providing a consistent explanation for the results observed in all three test methods [20,25]. In general, the main contributors to the antioxidant activity of DdEx are galloyl derivatives and ellagic acid, which, thanks to their specific chemical properties, participate effectively in neutralising free radicals and reducing ferric complexes.

The observed antibacterial activity of the ethanolic extract from the alga *D. dichotoma* was significant compared to the antibiotic (positive control) ampicillin, which was generally ineffective against most of the strains tested, with no inhibition zones or bactericidal effects observed. Thus, our present results clearly document the development and improvement of antibacterial resistance by the bacterial strains, which is consistent with previous studies [26,27,28]. Furthermore, our results are in agreement with those reported elsewhere [27,28], which showed that plants extracted from *Artemisia negrei* and *Artemisia flahaulti* possessed significant antibacterial powers against *E. coli* 57 and *E. coli* 97 and *S. aureus*. However, several studies have demonstrated and confirmed the ability of *D. dichotoma* medicinal algae to combat microbes. Our results are consistent with those of Mohammed Imran [29] who showed that *S. aureus* was recorded as the most sensitive organism, followed by *E. coli*, while *P. aeruginosa* showed the lowest inhibition at all concentrations. The antibacterial capacity of our ethanolic extract of *D. dichotoma* algae could be due to major compounds such as HHDP-digalloyl-glucose and its derivatives identified by HPLC-MS, such as galloyl glucoside and tetragalloyl-glucose, as well as minor compounds such as apigenin-7-O-glucoside and the apigenin-7-O-glucoside isomer. This hypothesis has been confirmed by previous studies [30].

Many studies have been devoted to the control of *S. cerevisiae* and *C. albicans* using natural products, in particular the study by El Moussaoui et al. [31], who showed that the plant *Withania frutescens* (L.) had antifungal activity against *S. cerevisiae* and *C. albicans*. Chebbac’s [26] study also reported that plants of the genus Artemisia had strong antifungal activity against *C. albicans*.

The presence of compounds possessing phenolic groups is known to exhibit promising biological activities, such as antimicrobial activities [32]. The presence of lipid molecules in *D. dichotoma* as indicated by FT-IR spectra may correlate with the observed antimicrobial activity; according to Fischer’s study [33], certain fatty acids may even be selective. Many algae are known to produce halogen-containing compounds, which is consistent with the chlorine and iodine functional groups detected in *D. dichotoma* extracts. Halogenated compounds have shown antimicrobial activities in previous studies [34].

The antimicrobial compound found in seaweed (*Dictyota acutiloba*) has been shown in another study to have a more powerful antagonistic effect against Gram-positive bacterial pathogens [35]. Furthermore, they demonstrated a strong antimicrobial effect on the bacteria *B. subtilis*. Furthermore, Demirel et al. [36] found that brown algal extracts exhibited a comparable order of activity; however, their crude extracts differed from the ones utilized in this investigation. It has been demonstrated that the brown seaweed *Fucus vesiculosus* contains an antibacterial compound called polyhydroxylated fucofloretol that is effective against both Gram-positive and Gram-negative bacteria. Variations in the makeup and structure of a specific bacterial group’s cell walls led to their susceptibility [37,38]. However, the outer membrane of Gram-negative bacteria blocks the effects of drugs and other environmental contaminants. This could be because antibiotics function as competitive inhibitors of the transpeptidase required by bacteria (primarily Gram-negative bacteria) to form a cell wall after they have penetrated their outer membrane through porins. This ultimately results in cell lysis and reduces the capacity of pathogens to replicate successfully.

According to the current data, the seaweed extract from *D. dichotoma* exhibits nearly comparable antibacterial activity against both Gram-positive and Gram-negative bacteria. Hence, we were able to verify that the algae extract from *D. dichotoma* can be a potentially effective tool in the fight against pathogenic strains that are resistant to drugs and nosocomial infections.

## 4. Materials and Methods

### 4.1. Algal Material

**Figure 5:**
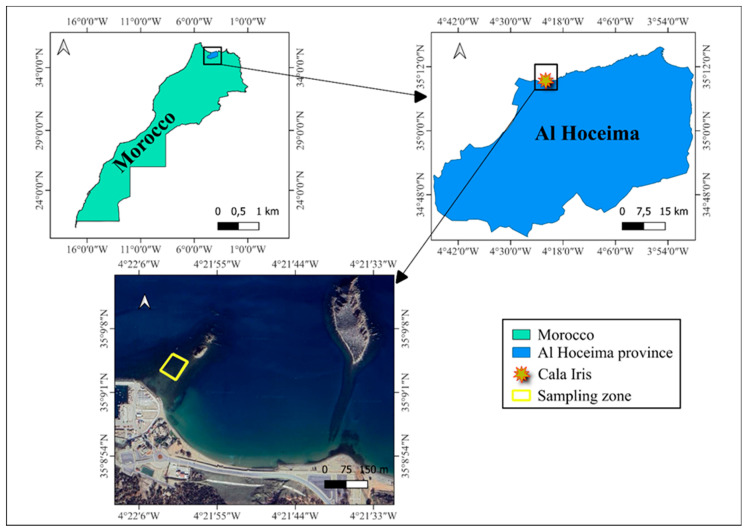
Map of sampling location (Cala Iris, Al Hoceima, Morocco).

*Dictyota dichotoma* was handpicked from the littoral zone of Al Hoceima (35°09′04.7″ N 4°22′00.7″ W) (Figure 5), during low tide in June 2023. The sample was carefully washed with seawater. Afterwards, the algae were transported to the laboratory and carefully washed with tap water to remove any sand debris or epibionts. After sun drying for a week, the algal biomass was ground to a fine powder less than 0.2 mm using a powder mill and stored at −20 °C for future use.

### 4.2. Crude Extract Preparation

The extraction process was carried out as described by Jawhari [39] with a few modifications. However, 6 g of algae powder was added to 60 mL of 70% (*v*/*v*) ethanol. The mixture was left overnight at room temperature under gentle shaking. Afterwards, the mixture was filtered through Whatman paper and the extract obtained was placed in an oven at 40 °C until complete evaporation of the liquid. The dried extract was stored at 4 °C.

### 4.3. Phytochemical Characterisation by HPLC-MS

The organic extract of *D. dichotoma* was analyzed by reverse-phase high-performance liquid chromatography (HPLC), using a diode array detector (DAD). Analysis was performed using a MOS-1 HYPERSIL analytical column (250 mm × 4.6 mm) packed with Exsil ODS (5 µm), coupled to a Thermo Scientific HPLC system. Compounds were separated in gradient mode using two solvents: A (water) and C (acetonitrile). The elution program was structured as follows: 80% solvent A and 20% solvent C for 1 min, followed by 60% solvent A and 40% solvent C for 2.5 min, then back to 80% solvent A and 20% solvent C for 4 min. The injection volume was set at 5 µL, with a constant flow rate of 1 mL/min. Phenolic compounds were identified on the basis of their retention times and UV spectra [40,41].

### 4.4. Evaluation of the Antioxidant Power of Dictyota dichotoma Extract

The antioxidant capacity of the DdEx sample was determined using three complementary methods: the DPPH method, the FRAP method and the phosphomolybdate (TAC) method. These techniques make it possible to assess the efficiency of the sample in neutralising free radicals, reducing ferric ions and its total antioxidant capacity, respectively, thus providing a complete characterisation of its antioxidant potential.

#### 4.4.1. DPPH Method

DPPH radical scavenging activity was measured according to the protocol described by LopesLutz et al. in 2008 [42]. A volume of 25 μL of each DdEx methanolic solution at different concentrations was added to 975 µL of DPPH methanolic solution (2.4 mg/100 mL methanol). In parallel, a negative control was prepared by mixing 25 μL of methanol with 975 mL of the methanolic DPPH solution. After vortexing, the tubes were placed in the dark at room temperature for 30 min. Readings were taken by measuring absorbance at 517 nm. The positive control was a solution of the standard antioxidant BHT. The results can be expressed as anti-free radical activity or free radical inhibition in percentages (I%) using the following formula:

(1) $$Inhibition\;\left(\%\right)=\left(1-\frac{Abs\;of\;sample}{Abs\;of\;negative\;control}\right)\times \;100$$

#### 4.4.2. FRAP Method

The reducing power of DdEx was determined using the Oyaizu method. First, 2.5 mL of a 0.2 M phosphate-buffer solution (pH 6.6) and 2.5 mL of a 1% potassium ferricyanide (K_3_Fe(CN)_6_) solution were added to tubes containing 1 mL of DdEx solutions at different concentrations [43]. The resulting mixtures were incubated at 50 °C for 20 min. Next, 2.5 mL of 10% trichloroacetic acid was added to stop the reaction. Finally, 2.5 mL of the reaction mixture was mixed with 2.5 mL of distilled water and 0.5 mL of a freshly prepared 0.1% FeCl3 aqueous solution. The absorbance of the reaction medium was read at 700 nm. BHT was used as a positive control.

#### 4.4.3. TAC Method

The total antioxidant capacity of DdEx was assessed using a method based on the reduction of the phosphomolybdenum complex [41,44]. To achieve this, 25 µL of DdEx was carefully mixed with 1 mL of a reagent solution composed of three reagents: sulphuric acid (0.6 M), sodium phosphate (28 mM) and ammonium molybdate (4 mM). This mixture was then incubated at 95 °C for 90 min to form a stable colour complex. After incubation, the absorbance of the solution was measured at a wavelength of 695 nm, corresponding to the absorption maximum of the complex formed. The absorbance intensity is directly proportional to the antioxidant capacity of the sample. The results were expressed in microgram of ascorbic acid equivalent per milligram of crude extract (µg AAE/mg), providing an accurate quantification of antioxidant activity.

### 4.5. Evaluation of Antimicrobial Activity by Solid-State Diffusion

The antimicrobial activity of the DdEx sample was assessed using the solid-state diffusion test. Microbial strains selected for this study included *S. aureus*, *E. coli*, *K. pneumoniae*, *P. mirabilis*, *C. albicans* and *S. cerevisiae*. These microorganisms were grown in Mueller–Hinton (MH) and malt extract (ME) media, respectively. The fresh cultures obtained in MH and ME media were then diluted decimally in sterile saline (0.1% NaCl), to obtain a final inoculum concentration of between 10^6^ and 10^8^ CFU/mL. The microbial suspensions thus prepared were used to inoculate Petri dishes containing the appropriate culture media. After inoculation, 20 μL of the DdEx (2 mg/mL; DMSO 5%) sample was applied to the plates. In parallel, standard antibiotics, such as ampecilin (AMP) for bacteria and fluconazole (Flu) for fungi, were used as positive controls. The antimicrobial activity of the DdEx sample was determined by measuring the zone of inhibition (in mm) around the application points, providing a direct comparison with standard antimicrobial agents [45,46].

### 4.6. Molecular Docking

Molecular docking was used to assess the potential antioxidant and antimicrobial effects of compounds identified in the extract of *Dictyota dichotoma* (DdEx). Ligands were prepared from compounds obtained via PubChem, using the LigPrep module of Schrödinger software (version 11.5) and the OPLS3 force field. Ionization was optimized at pH 7.0 ± 2.0, generating up to 32 stereoisomers per molecule. Target proteins, such as human NADPH oxidase and other bacterial and fungal enzymes, were extracted from the Protein Data Bank and refined by adding hydrogen atoms, correcting bond orders, removing water molecules, and minimizing energy. Flexible docking was performed in Standard Precision (SP) mode in Glide, with a penalty for non-cis/trans amide bonds, adjustments for Van der Waals interactions, and analysis based on the glide score, allowing the selection of the pose with the lowest energy for each ligand [41,47].

### 4.7. Statistical Analysis

Statistical analysis was carried out using GraphPad Prism 8 software, developed by Microsoft (San Diego, CA, USA), to calculate means and standard deviations.

## 5. Conclusions

This study highlighted the therapeutic potential of the ethanolic extract of *Dictyota dichotoma*, demonstrating both its antioxidant and antimicrobial capacities. In vitro and in silico biological potency assessment revealed that this seaweed is a valuable source of bioactive compounds that can be used as natural antioxidants and antimicrobials. Phytochemical characterization by HPLC-MS identified a diversity of phycocompounds, mainly phenols, which are likely to be responsible for these biological activities. In vitro tests confirmed significant antioxidant power, while antimicrobial evaluations showed significant efficacy against various bacterial and fungal strains. In addition, the in silico approach identified the most promising compounds in terms of potential mechanisms of action, paving the way for future research into the development of new natural therapeutic agents. These results underline the importance of *Dictyota dichotoma* as a promising source of bioactive compounds, warranting further investigation into its potential applications in pharmacology and other health-related fields.

## Figures and Tables

**Table 1:** Results of phytochemical analysis of *Dictyota dichotoma* extract by HPLC-MS.

No	RT	*m*/*z* (M-H)-	Fragments	Proposed Compounds	Concentration (µg/mg of DdEx)
**1**	0.31	785.1	301/225	HHDP-digalloyl-glucose	20.12
**2**	9.48	357	289	nd	0.37
**3**	9.65	361	359/112	Secoisolariciresinol	5.36
**4**	15.98	331	289/91	Galloyl-glucoside	6.94
**5**	17.47	331	289/91	Gallolyl-hexoside	8.74
**6**	17.65	531.1	361	nd	3.47
**7**	25.35	783.1	480/301	Tetragalloyl-glucose	4.18
**8**	25.75	433.12	407/385/301/265	Ellagic acid-pentose	5.13
**9**	25.84	483.12	313	Digalloyl- glucoside	5.41
**10**	25.9	939.1	787/769	Pentagalloyl-glucose	6.34
**11**	26.24	407.2	401/28	nd	6.3
**12**	26.4	431.7	269.1/125	Apigenin-7-O-glucoside	2.1
**13**	26.5	996.87	725/579	Punictannin B	4.83
**14**	26.67	389.5	289/90.1	nd	0.44
**15**	26.75	433.12	301.2/289.2/90.1	Pelargonidin 3-O-glucoside	0.11
**16**	27.03	447.12	401.25/289.1	Ellagic acid-deoxyhexose	4.39
**17**	27.18	394.1	301/265	Methylation and sulfation of ellagic acid	0.46
**18**	27.49	417.03	353.1/289.2	nd	4.36
**19**	27.7	289.1	91.02	catechine	3.48
**20**	27.8	431.12	269.1/90.1	Apigenin-7-O-glucoside isomer	2.11
**21**	28.1	787.1	745.1/607.2	Tetragalloyl-glucose	1.46
**22**	29.43	633.1	531.2/349.1	Galloyl-HHDP-glucoside	3.87

nd: not detected.

**Table 2:** Inhibition zones induced by the *D. dichotoma* extract and the control (ampicillin) against bacterial strains (mm) and the control fluconazole against two yeasts of the genus *Candida*.

	*E. coli*	*S. aureus*	*K. pneumoniae*	*P. mirabilis*	*C. albicans*	*S. cerevisiae*
DdEx	22.58 ± 0.58	11.93 ± 0.73	10.56 ± 1.48	18.81 ± 1.05	22.38 ± 1.24	23.52 ± 0.93
AMP	0.0 ± 0.0	0.0 ± 0.0	0.0 ± 0.0	0.0 ± 0.0	Nt	Nt
Flu	Nt	Nt	Nt	Nt	19.70 ± 0.40	18.81 ± 0.67
DMSO (5%)	0.0 ± 0.0	0.0 ± 0.0	0.0 ± 0.0	0.0 ± 0.0	0.0 ± 0.0	0.0 ± 0.0

AMP: ampicillin, Flu: fluconazole. Nt: non tested.

**Table 3:** Docking results with ligands in different receptors.

	Glide Gscore (Kcal/mol)			
	2CDU	1FJ4	3Q8U	5FSA
**Apigenin-7-O-glucoside**	−7.424	−7.276	−7.698	−9.569
**Catechin**	−5.55	−6.453	−6.796	−7.638
**Ellagic acid**	−5.114	−4.921	−7.935	−7.315
**Ellagic acid deoxyhexoside**	−5.749	−5.072	−6.76	−8.66
**HHDP-galloyl-glucose**	−5.185		−7.858	−5.251
**Pelargonidin 3-O-glucoside**	−5.465	−6.811	−7.244	−8.925
**Pentagalloylglucose**	−6.402		−10.502	−7.014
**Secoisolariciresinol**	−4.952	−6.594	−7.292	−8.441
**Tetragalloyl-glucose**	−7.723		−10.47	−8.778

## Data Availability

All study data are included in the article.
